# Transcriptional profiling of *Vibrio parahaemolyticus exsA* reveals a complex activation network for type III secretion

**DOI:** 10.3389/fmicb.2015.01089

**Published:** 2015-10-20

**Authors:** Aaron C. Liu, Nikhil A. Thomas

**Affiliations:** ^1^Department of Microbiology and Immunology, Dalhousie UniversityHalifax, NS, Canada; ^2^Department of Medicine (Infectious Diseases), Dalhousie UniversityHalifax, NS, Canada

**Keywords:** Type III secretion, transcription activator, gene regulation, environmental sensing

## Abstract

*Vibrio parahaemolyticus* (Vp) is a marine halophilic bacterium that is commonly associated with oysters and shrimp. Human consumption of contaminated shellfish can result in Vp mediated gastroenteritis and severe diarrheal disease. Vp encodes two type 3 secretion systems (T3SS-1 and T3SS2) that have been functionally implicated in cytotoxicity and enterotoxicity respectively. In this study, we profiled protein secretion and temporal promoter activities associated with *exsA* and *exsB* gene expression. *exsA* is an AraC-like transcriptional activator that is critical for activating multiple operons that encode T3SS-1 genes, whereas *exsB* is thought to encode an outer membrane pilotin component for T3SS-1. The *exsBA* genetic locus has two predicted promoter elements. The predicted *exsB* and *exsA* promoters were individually cloned upstream of *luxCDABE* genes in reporter plasmid constructs allowing for *in situ*, real-time quantitative light emission measurements under many growth conditions. Low calcium growth conditions supported maximal *exsB* and *exsA* promoter activation. *exsB* promoter activity exhibited high basal activity and resulted in an *exsBA* co-transcript. Furthermore, a separate proximal *exsA* promoter showed initial low basal activity yet eventually exceeded that of *exsB* and reached maximal levels after 2.5 h corresponding to an entry into early log phase. *exsA* promoter activity was significantly higher at 30°C than 37°C, which also coincided with increased secretion levels of specific T3SS-1 effector proteins. Lastly, bioinformatic analyses identified a putative expanded ExsA binding motif for multiple transcriptional operons. These findings suggest a two wave model of Vp T3SS-I induction that integrates two distinct promoter elements and environmental signals into a complex ExsA activation framework.

## Introduction

*Vibrio parahaemolyticus* (Vp) is a significant food borne human pathogen associated with severe diarrhea arising from enteric infections (Ceccarelli et al., 2014). It has also been associated to a lesser extent with wound infections and septicaemia. A pandemic serotype Vp O3:K6 has emerged, reaching many continents of the world, suggesting that this serotype has adapted to efficiently establish disease in humans (Velazquez-Roman et al., 2014). Sequence data from various Vp isolates indicate that many but not all human pathogenic strains contain two type III secretion systems (T3SS) (Makino et al., 2003; Banerjee et al., 2015). Studies using a small animal infection model with Vp strain RIMD2216330 (O3:K6) have demonstrated that both secretion systems function to deliver effector proteins into host cells, with T3SS-1 likely contributing to host cell cytotoxicity and T3SS-2 being involved in intestinal disruption and acute diarrhea (Ritchie et al., 2012). Therefore, it has been established that type III secretion is a key facet of Vp infection biology. In contrast, little is known regarding what environmental or host factors contribute to regulating T3SS genes and only a few studies have evaluated Vp T3SS gene regulation (Kodama et al., 2010a; Zhou et al., 2010a; Livny et al., 2014).

The majority of T3SS-1 associated genes within Vp are found in operon or co-transcribed units, similar to the situation found in other pathogens that express T3SS. Regulation of T3SS-1 genes in *V. parahaemolyticus* occurs in part through its transcriptional activator ExsA and a cognate anti-activator ExsD (Zhou et al., 2008), presumably functioning in a manner similar to the homologs that are found *in Pseudomonas aeruginosa* (King et al., 2013). ExsA is considered a member of the AraC/XylR transcriptional activator family and physically binds and bends DNA to activate gene transcription as demonstrated in both Vp and *P. aeruginosa* (Zhou et al., 2008; Brutinel et al., 2009). ExsA family members are thought to recognize and bind DNA via two intrinsic helix turn helix (HTH) domains. In the case of *P. aeruginosa*, two adjacent DNA binding sites are thought to support binding of two ExsA monomers (Diaz et al., 2011). Moreover, the monomers interact with their respective amino-terminal domains to form a functional ExsA homodimer. ExsD acts as an anti-activator, binding to an ExsA monomer within the cell, thereby preventing ExsA from binding to DNA and limiting ExsA dependent gene transcription (Zhou et al., 2010a; Kodama et al., 2010b). ExsA is likely involved in transcriptional activation of 8 or more operons which contain over 40 T3SS-1 associated genes, thereby placing it as a key activator of T3SS-1 expression.

Specific environmental signals that are sensed by pathogens often result in temporal and coordinated virulence gene expression. Temperature and low calcium has been implicated in inducing active type III secretion for a variety of pathogens (Fields et al., 1994; Thomas et al., 2005; Diaz et al., 2011), although the regulatory mechanisms that promote secretion events remain poorly understood. Many studies have demonstrated that low calcium growth conditions support the expression of the *P. aeruginosa* T3SS gene repertoire in an ExsA dependent manner (Diaz et al., 2011). Recently, a proteomic study revealed that low calcium growth conditions supported protein expression and secretion for many of the Vp T3SS-1 translocators and effectors (Sarty et al., 2012). It has also been reported that Vp *exsA* gene expression was upregulated when Vp was in contact with HeLa cells, indicating that T3SS-1 gene expression is induced during infection-like conditions (Zhou et al., 2008). Moreover, low cell density has been observed to promote Vp and *V. harveyi* T3SS-1 gene expression via quorum sensing mechanisms (Henke and Bassler, 2004). In contrast, *exsA* gene expression is inhibited by the global negative regulator H-NS (Sun et al., 2015). Still, environmental signals and requisite genetic mechanisms that result in *exsA* gene transcription, and hence T3SS-1 activation, remain unresolved.

In this study we set out to evaluate what factors contribute to T3SS-1 expression. Based on previous studies that evaluated T3SS-1 protein expression (Park et al., 2004b; Ono et al., 2006; Sarty et al., 2012), we assayed *in situ* real-time *exsB* and *exsA* promoter activity under various culture conditions which revealed differential activation patterns. Total secreted protein profiles for tested cultured conditions further revealed differential secretion of translocators and effectors. The data suggest a two wave model of Vp T3SS-1 induction that integrates environmental signals into a complex ExsA activation framework.

## Materials and methods

### Bacterial strains and growth conditions

Bacterial strains generated and used in this study are listed in Table 1. Bacteria were routinely cultured in Luria broth (LB) [Sigma, L3522] at 37°C with shaking at 200 r.p.m., or 30°C at 250 r.p.m. for specific assays. Antibiotics (Sigma) were added when appropriate, to a final concentration of 100 μg kanamycin ml^−1^ (for Vp strains) or 50 μg for *Escherichia coli* DH5α. Sterile media supplements (MgSO_4_, EGTA, CaCl_2_) were added to basal LB media from concentrated stock solutions followed by a brief 10 min equilibration prior to use in assays.

**Table 1:** **Bacterial strains and plasmids used in this study**.

	**Description**	**Source/Reference**
**STRAIN**		
*Vibrio parahaemolyticus* O3:K6 RIMD 2210633	Wild type *Vibrio parahaemolyticus*	Makino et al., 2003
Vp Δ*vscN1*	Non-functional T3SS1	Sarty et al., 2012
Vp Δ*vscN1*/Δ*vscN2*	Non-functional T3SS1 and T3SS2	Sarty et al., 2012
DH5α	*E. coli* cloning strain	
**PLASMID**		
pJW15	Vector containing promoter-less *luxCDABE*	Macritchie et al., 2008
p*exsA*-lux	*exsA* promoter cloned into pJW15 with	This study
p*exsB*-lux	*exsB* promoter cloned into pJW15 with	This study
p*exsD*-lux	*exsD* promoter cloned into pJW15	This study
p*rplN*-lux	*exsD* promoter cloned into pJW15	This study
pFLAG-CTC	Plasmid used to incorporate tac promoter and C-terminal FLAG tag to cloned DNA	Sigma
pVSV105	Vibrio specific vector, replicates in Vp and DH5al*pir*	Eric Stabb
p*exsA*-FLAG	Expresses ExsA-FLAG from a *tac* promoter	This study

### Recombinant DNA techniques

All PCR, cloning, and recombinant DNA techniques were performed using standard protocols. Routine DNA cloning steps were performed in DH5α, followed by introducing relevant recombinant plasmids into Vp using electroporation. Restriction endonucleases and DNA polymerases were purchased from New England Biolabs (NEB). Oligonucleotide primers were obtained from Integrated DNA Technologies (IDT) (Table 2). pJW15 contains a promoter-less *luxCDABE* cassette (Macritchie et al., 2008) and served as a vector for cloning *exsA, exsB, exsD, and rplN* promoter fragments. To generate a recombinant ExsA-FLAG expression plasmid, Vp *exsA* (without a stop codon) was amplified from chromosomal DNA by PCR using primers NT387 and NT388 followed by cloning into pFLAG-CTC. A PCR using primers NT139 and NT140 and *exsA*/pFLAG-CTC as DNA template yielded a product containing a 5′ *tac* promoter and a 3′ FLAG tag fused to *exsA*. The product was finally blunt end cloned into pVSV105 and the resulting plasmid was introduced into Vp by conjugal mating. All plasmid constructs were subjected to DNA sequence analyses to verify cloned DNA.

**Table 2:** **Oligonucleotides used in this study**.

**Title**	**Sequence**	
NT337	CCGAATTCAATCGGTTACATTTAATTAGCGC	p*exsA*-lux
NT339	CCGGATCCCGTTTCTGTGTTTAGTTGGCCTG	pexsA-lux
AL397	CCGAATTCGCGTGCTGACATAGAAACAGTCCT	p*exsB*-lux
AL398	GCGGATCCGACTGAGCGCATCCAGTCAATAAC	p*exsB*-lux
AL399	TCCGTTCAGAGCCAAGCGAATGTAT	RT-PCR
AL400	CTGCGATAGCAAGGCATAGAGGACT	RT-PCR
AL412	TTGAATTCGTGATGAGCAAGTCGCCATCGCGA	p*exsD*-lux
AL414	ATTGGATCCTGCGTCACAATGCTGTCAGGA	p*exsD*-lux
AL415	TTGAATTCAAGGCGAAGATGTAATCTTCACTC	p*rlpN*-lux
AL416	AAGGATCCATCAGCTGCGTCCAGCATACTT	p*rlpN*-lux
NT139	CATCATAACGGTTCTGGCAAATATTC	pFLAG-CTC
NT140	CTGTATCAGGCTGAAAATCTTCTCTC	pFLAG-CTC
NT387	AAACCGCATATGGATGTGTCAGGCCAACTAAACAC	*exsA*
NT388	CCGGTACCATTCGCGATGGCGACTTGCTCATCACC	*exsA*

### Reverse transcriptase (RT)-PCR

Total RNA was isolated from Vp using a Qiagen RNeasy kit. RNA preparations were treated with DNase and then column purified. Reverse transcription reactions using primer AL400 were performed with Omniscript reverse transcriptase (Qiagen) using the protocol described by the manufacturer. A PCR reaction using cDNA template generated from the reverse step was performed using primers AL399 and AL400. A separate PCR using Vp genomic DNA was used to generate the predicted amplicon control of 1.5 kb. The amplicon spans the partial open reading frame of *exsB* to within *exsA*, including the intergenic region.

### Luciferase (lux) reporter assays

Luciferase reporter assays were performed similar to a previous promoter study (Brouwers et al., 2012). End-point or multiple point assays were performed with Vp strains harboring lux plasmid constructs. For end-point assays, starting cultures (OD_595_ 0.025) were grown for 2.5 h in LB supplemented with magnesium and ethylene glycol tetracetic acid (Mg+EGTA) followed by a OD_595_ measurement by a Biophotometer Plus (Eppendorf) at a path length of 10 mm. The samples were then aliquoted into clear bottom white walled multiwell plates (Costar #3610) and measured for light emission by counts per second (c.p.s.) using a Victor X5 instrument (Perkin Elmer). For multiple time point assays, the relevant samples were initially prepared in the same way as for the end-point assays, followed by immediate OD_595_ and cps reading at each time point. The data were subjected to a Two Way ANOVA with a standard Tukey test. Plotted data is the average from three experiments. Data was plotted using GraphPad Prism5 software.

### Protein secretion assays

A secretion assay protocol using syringe end filters was used to produce total secreted protein fractions from Vp culture supernatants (Sarty et al., 2012). Briefly, bacterial cultures (starting OD_595_ 0.025, 30°C, 250 rpm) were harvested after 4 h of growth. Three milliliters of culture was centrifuged to remove whole cells, followed by filtering the clarified supernatant through a 0.22 μm PVDF syringe-end filter (Millipore). The resulting culture supernatant was subjected to trichloracetic acid precipitation [10% (vol/vol)] on ice. The sample was centrifuged at 16,000 × *g* followed by resuspending the pellet in 10 μl electrophoretic sample loading buffer (2XESB) as previously described (Sarty et al., 2012).

### Protein electrophoresis

All protein samples were separated by SDS-PAGE as described elsewhere (Laemmli, 1970). Separated polypeptides were visualized by Coomassie G-250 blue staining.

## Results

### Evaluation of *exsA* and *exsB* promoter activities

**Figure 1:**
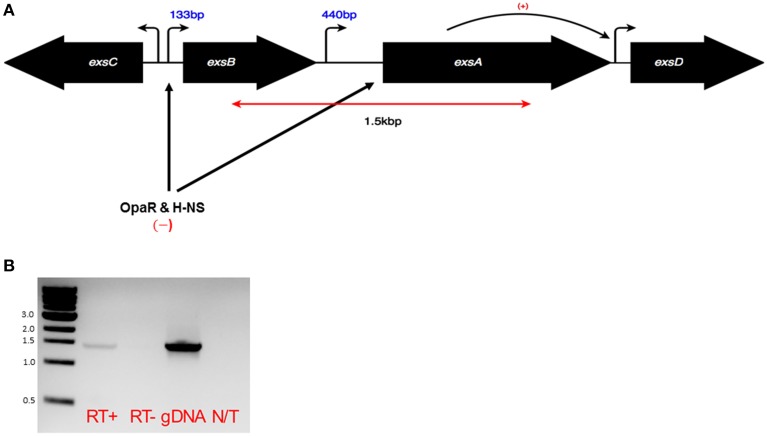
**(A)** Schematic of the *exsA* gene locus and adjacent genes. Bent arrows indicate promoter elements. In the case of *exsB* and *exsA* mapped transcriptional start sites are known, with numbers indicating mRNA leader lengths. The double headed red arrow corresponds to the predicted product size of the co-transcript as shown in a RT-PCR experiment **(B)**. Black arrows indicate the roles of specific transcriptional regulators acting at indicated sites. **(B)** RT-PCR using sequence specific primers and purified mRNA was carried out to evaluate the existence of an *exsBA* co-transcript. Genomic DNA was used as a template to show primer specificity and expected amplicon length. The RT-PCR amplified 1.5 kb region is denoted in **(A)**.

The Vp *exsBA* genetic locus has two promoter elements that support transcription initiation upstream of *exsB* and *exsA* respectively (Sun et al., 2015) (Figure 1A). In *V. harveyi*, a multigene *exsBA* transcript has been detected, presumably driven from the *exsB* promoter (Waters et al., 2010). This observation is somewhat surprising given the long intergenic region between *exsB* and *exsA*, in addition to the presence of gene specific promoters. Therefore, we set out to determine if Vp *exsBA* are co-transcribed. As shown in Figure 1B, Vp generated an *exsBA* mRNA co-transcript as determined by reverse transcription PCR (RT-PCR).

To investigate individual *exsB* and *exsA* promoters, the relevant chromosomal DNA fragments were fused to a promoterless *luxCDABE* gene cassette within pJW15 (Table 1). Plasmids p*exsA*-lux, p*exsB*-lux and pJW15 were each electroporated into wild type Vp. Notably, the p*exsB*-lux and pexsA-lux transformant colonies recovered post- electroporation were observed to emit light when grown on solid LB agar, whereas the transformants harboring pJW15 did not emit light (data not shown). This indicated that the cloned *exsB and exsA* promoters were functional and supported transcriptional activity when Vp was grown on LB.

**Figure 2:**
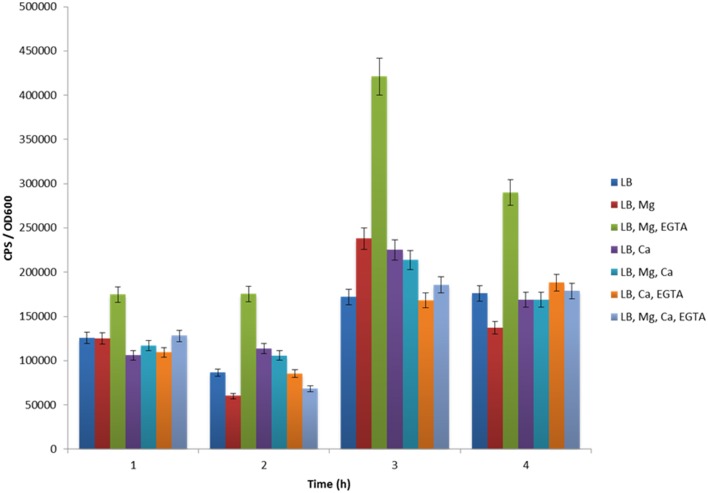
**The effect of various growth media formulations on ***exsA*** promoter activity**. Maximal *exsA* promoter activity was evident when Vp was grown in LB+Mg+EGTA for all time points assessed.

ExsA is known to act at many Vp T3SS-1 operons, including those involved in forming secretion apparatus components (Zhou et al., 2008). Using Vp harboring p*exsA*-lux, we set out to evaluate *exsA* gene expression under a variety of media conditions at 30°C. This growth temperature was previously shown to support high levels of translocon and effector protein secretion via T3SS-I (Sarty et al., 2012). We show here that LB supports low level *exsB* and *exsA* promoter activity therefore we used it as the derivation point for the basal growth media. In luciferase (lux) assay experiments, real-time *in situ exsA* promoter activation is directly proportional to light emitted from the bacterial culture (Meighen, 1993). When Vp with p*exsA*-lux was grown in LB for 1 h, a basal level of *exsA* promoter activation was observed (Figure 2), consistent with the light emitting colonies observed when Vp was grown on LB agar. Vp *exsA* promoter activity for LB supplemented with magnesium or calcium was comparable to LB alone, however, LB supplemented with magnesium and EGTA produced significantly elevated *exsA* promoter activation (Figure 2). *exsA* promoter activation was even more pronounced after 3 h. Bacteria continued to demonstrate *exsA* promoter activation in LB supplemented with magnesium and EGTA after 4 h of incubation, although promoter activity exhibited a decreasing trend from that observed after 3 h. The data indicate that LB containing magnesium and EGTA supported maximal *exsA* and *exsB* promoter activation. LB that contained magnesium, calcium, or both elements, all supported basal level activation comparable to LB (only). Notably, the addition of calcium to LB media containing magnesium and EGTA lowered *exsA* and *exsB* promoter activation (over all time points evaluated) implicating calcium sensing in T3SS-1 gene expression (see Discussion).

**Figure 3:**
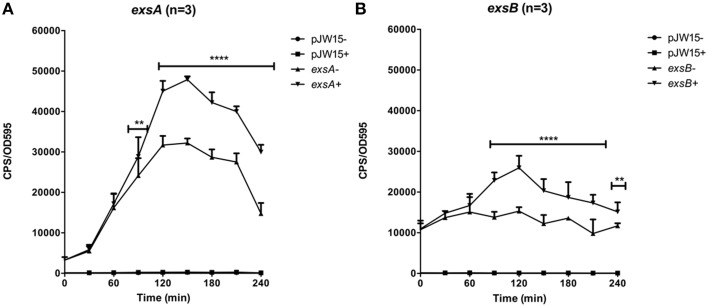
**Temporal promoter profiles for (A) ***exsA*** and (B) ***exsB*** were generated by diluting overnight cultures containing the reporter plasmids in LB medium (–) and LB supplemented with EGTA, MgSO4 (+)**. Real time measurements were collected at 30 min intervals and normalized to OD595. The averaged data values from three experiments are shown. Error bars represent S.E.M. ^**^p < 0.01, ^****^p < 0.0001. Representative growth curves for each strain from a single experiment are shown below the relevant data set.

Experiments to evaluate *exsB* promoter activation revealed a similar trend to that observed for the *exsA* promoter (Figure 3). One notable difference was that *exsB* promoter activity was higher (10000–12000 units) than that of exsA (5000–7000 units) at early time points (0–30 min). Maximal *exsA* and *exsB* promoter activation occurred at 2.5 h in growth media containing Mg+EGTA. This time point also corresponded to when the bacteria were entering into early log phase growth (Figure 3). The effect of growth media on *exsB* promoter activation was similar to that observed for *exsA* promoter activation (Figure S1). Collectively, these data quantitatively demonstrate differential activity profiles for *exsB* and *exsA*.

### T3SS-1 activity is not required for Vp *exsA* gene expression

**Figure 4:**
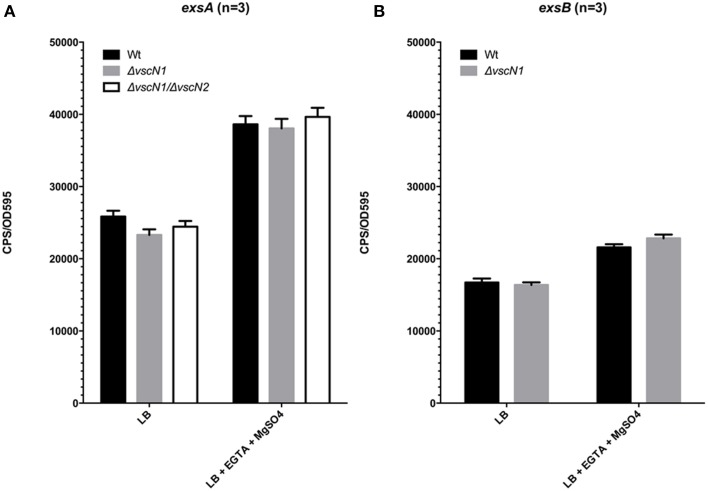
**T3SS-1 and T3SS-2 activity are not required for ***exsA*** or ***exsB*** promoter activation**. **(A)** *exsA* promoter activity in wild type Vp, Δ*vscN1* (T3SS-1 ATPase-deficient mutant), and Δ*vscN1*Δ*vscN2*. **(B)** *exsB* promoter activity in wild type Vp and Δ*vscN1*. Endpoint measurements were taken at 2.5 h and compared. The averaged data values from three experiments are shown. Error bars represent S.E.M.

For many pathogens with a T3SS, a negative regulator protein is often secreted through the assembled T3SS apparatus. This action typically leads to effector gene expression. For Vp, the negative regulator ExsE interacts with ExsC within the cytoplasm, which allows ExsD to inhibit ExsA activity. Upon secretion of ExsE by the T3SS-1, ExsC interacts with ExsD, thereby allowing ExsA to activate T3SS-1 associated genes (Kodama et al., 2010b). To assess whether ExsE secretion impacts on *exsA* promoter activity, the p*exsA*-lux construct was introduced into Vp Δ*vscN* and Vp Δ*vscN*1Δ*vscN*2. These mutants are defective for T3SS-1 and T3SS-1 and T3SS-2 respectively due to the absence of ATPases normally required for function. As shown in Figure 4A, *exsA* promoter activity was unchanged in Δ*vscN* and Δ*vscN*1Δ*vscN*2 compared to the activity observed for wild type. Furthermore, we profiled *exsA* promoter activity within Δ*vscN* over 4 h and did not see any change compared to wild type Vp (Figure S1). Furthermore, *exsB* promoter activity was unchanged in Δ*vscN* (Figure 4B). These data indicate that under the conditions tested, the absence of T3SS-1 or T3SS-2 activity does not significantly impact on *exsA* promoter activation. ExsB is thought to encode a T3SS-1 pilotin and the data indicates that T3SS-1 activity does not impact on *exsB* promoter activity.

### *exsA* promoter activity is auto-activated

**Figure 5:**
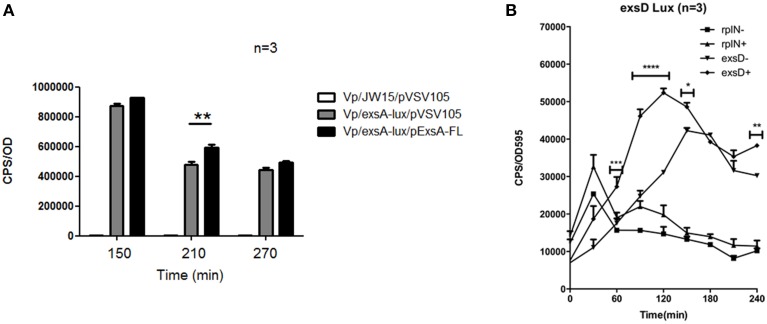
**ExsA is involved in auto-activation and mediates ***exsD*** expression**. **(A)** The effect of ExsA overexpression on *exsA* promoter activity was assessed using a lux assay. Error bars represent S.E.M, ^**^p = 0.015. **(B)** Temporal promoter activity profiles for *exsD* and *rplN* were generated by diluting overnight cultures containing the reporter plasmids in LB medium (−), and LB supplemented with EGTA, MgSO4 (+). Real time measurements were collected at 30 min intervals and normalized to OD595. The averaged data values from three experiments are shown. Error bars represent S.E.M. ^*^p < 0.05, ^**^p < 0.01, ^****^p < 0.0001 for comparison of *exsD-lux* conditions (±).

A common feature of AraC family regulators is the ability to auto-activate gene expression. To investigate this possibility for Vp ExsA, we generated a plasmid encoded ExsA-FLAG expression construct, with its transcription regulated by a *tac* promoter. This plasmid is expected to deliver ExsA overexpression without influences of feedback regulation or quorum sensing (due to the absence of the native *exsA* promoter). The ExsA-FLAG expression plasmid or empty control plasmid was transferred to Vp harboring p*exsA*-lux via conjugation. The resulting bacterial strains were then subjected to lux assays. ExsA overexpression did not impact *exsA* promoter activity when bacteria were grown in LB supplemented with magnesium +EGTA (data not shown). When bacteria overexpressing ExsA were grown in LB only, *exsA* promoter activity increased after 2.5 h compared to bacteria expressing native levels of ExsA. The increase reached statistical significance after 3.5 h (*p* = 0.015) and continued to 4.5 h (Figure 5A). These data indicate that ExsA overexpression results in higher levels of *exsA* promoter activity. Furthermore, in conditions that typically support low levels of type III protein secretion (LB growth), ExsA overexpression was observed to upregulate *exsA* promoter activity. This result is in complete agreement with a previous observation showing increased T3SS-1 associated gene expression upon ExsA overexpression (see Discussion).

### *exsD* promoter activity is higher in conditions that support maximal *exsA* gene expression

In Vp, ExsD binds to ExsA within the cell, thereby preventing ExsA binding to DNA (Zhou et al., 2010a). This regulatory paradigm is also observed in *P. aeruginosa*, where *exsACDE* are co-transcribed as a single operon. Hence, in *P. aeruginosa* the transcriptional activator ExsA is co-regulated with its own inhibitor. In Vp, the gene organization is different from *P. aeruginosa*. Specifically, *exsD* has its own promoter (Figure 1). Therefore, we set out to investigate whether the *exsD* promoter would show a similar activity profile to that of the *exsA* promoter. To address this we generated a p*exsD*-lux plasmid. We also generated a p*rplN*-lux plasmid as a control since *rplN* is a ribosomal protein gene that is not expected to be regulated by ExsA. These plasmids were added to wild type Vp and the bacteria were subjected to lux assays. As shown in Figure 5B, *exsD* promoter activity reached a maximal level at 2 h. Importantly, this occurred in growth conditions that supported maximal *exsA* promoter activity (LB containing Mg+EGTA, refer to Figure 2). When bacteria were grown in LB only, *exsD* promoter activity increased, although at a slower rate than when bacteria were grown in LB supplemented with Mg+EGTA. The *rplN* promoter showed an early sharp increase in activity irrespective of growth media (Figure 5B). This was expected since the bacteria had been introduced into fresh growth media and ribosomal protein gene expression would be required to support *de novo* protein synthesis. More importantly, *rplN* promoter activity showed a steady state temporal activity trend between 60 and 240 min which was different to the activity profile for the *exsD* promoter. These data reveal that *exsD* promoter activity correlates with that of *exsA*.

### Growth of Vp in media supplemented with magnesium, calcium, or EGTA produce differential secreted protein profiles

We previously reported growth conditions that support maximal levels of Vp translocator and effector secretion (Sarty et al., 2012). Based on the differential *exsA* promoter activation patterns in several distinct media (Figure 2), we reassessed the data and provide new observations. For these experiments, Vp was grown for 4 h and filtered culture supernatants were processed for SDS-PAGE and Coomassie staining (see Materials and Methods).

**Figure 6:**
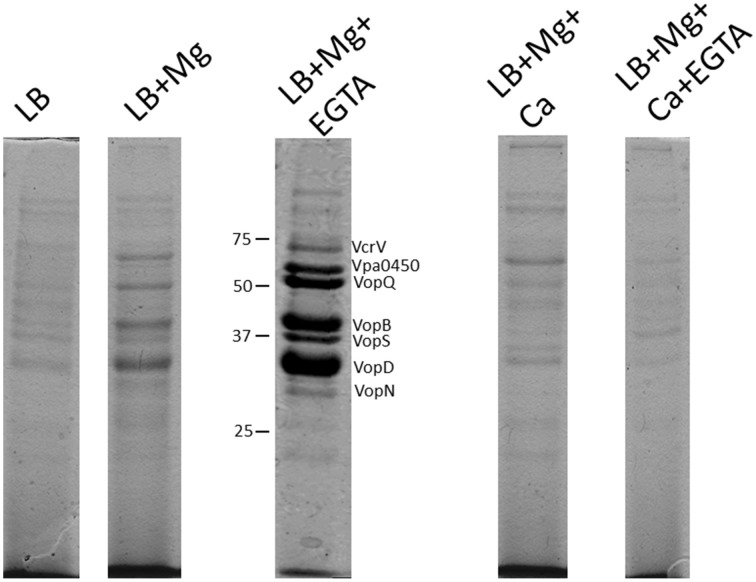
**Total protein secretion profiles of Vp grown for 4 h in the indicated growth media conditions**. Samples were normalized for cell density to account for growth rate differences. All protein samples were stained with Coomassie G-250. The protein species have been previously identified using mass spectrometry approaches. Note the specific appearance of Vpa0450 and VopN under LB+Mg+EGTA conditions only. The addition of calcium to media containing Mg+EGTA reduced the secretion of all T3SS-1 associated proteins.

Vp was grown in LB media with different supplementations, similar to the lux based *exsA* promoter activation studies. It was noted that each media produced differential total secreted protein profiles (Figure 6). The addition of EGTA to LB reduced Vp growth rate (data not shown), probably as a result of cell envelope stress and hence the secreted protein profile was too complex to interpret. Vp grown in LB had a complex secreted protein profile, however, the addition of magnesium to LB resulted in the appearance of 4 dominant protein species. Furthermore, growth of Vp in LB media containing magnesium and EGTA produced additional dominant secreted proteins (Figure 6). In contrast, growth of Vp in LB supplemented with (i) magnesium and calcium or (ii) magnesium, calcium and EGTA, did not yield abundant levels of these proteins.

The secreted proteins produced from Vp growth in LB+Mg+EGTA were previously subjected to mass spectrometry analyses and identified as type III translocators and effector proteins (Sarty et al., 2012). Notably, VopN and Vpa450 secretion were readily apparent in Vp cultures supplemented with Mg+EGTA yet not detectable from Vp cultures supplemented with magnesium (only) or any media supplemented with calcium (Figure 6). This observation appeared unique to VopN and Vpa0450. These data indicate that defined growth conditions significantly impact the T3SS-1 associated secretion profile of Vp and that certain effectors are efficiently secreted under low calcium conditions.

### Growth temperature impacts on *exsA* promoter activity and T3SS-1 secretion levels

**Figure 7:**
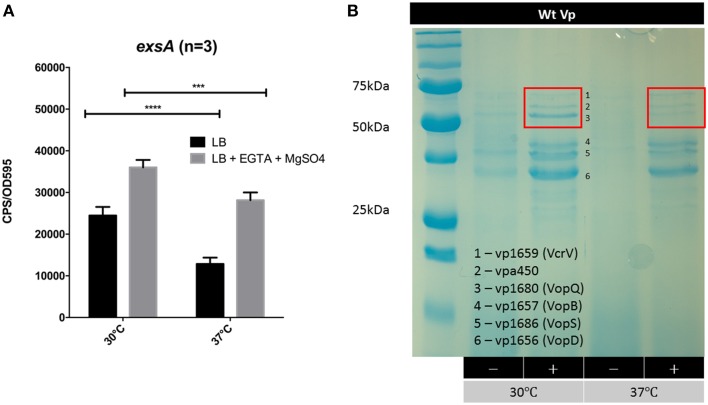
**The effect of growth temperature on ***exsA*** promoter activity and Vp total secretion profiles**. **(A)** *exsA* promoter activity measured in LB (−) or LB+Mg+EGTA (+) grown at 30°C or 37°C. Measurements were taken at 2.5 h post inoculation and normalized to OD595. ^***^p < 0.001, ^****^p < 0.0001. **(B)** Total secretion profiles revealed by Coomassie G-250 staining. Vp was grown in LB (−) or LB+Mg+EGTA (+), at 30°C or 37°C. Culture supernatants were harvested 4 h post inoculation. The red boxes highlight different banding intensities of effector proteins between the two conditions.

Vp is commonly found in brackish estuarine waters that range in temperature from 15 to 30°C (Ceccarelli et al., 2014). However, during human infection, Vp encounters a body temperature of 37°C. We set out to assess *exsA* promoter activity at 30°C and 37°C. It was observed that *exsA* promoter activity was significantly higher at 30°C when compared to 37°C (Figure 7A). The culture supernatants from these samples were subjected to SDS-PAGE to assess total protein secretion under the different temperature conditions. Coomassie staining revealed that effector protein secretion was more abundant at 30°C than 37°C (Figure 7B). These data correlated with the elevated *exsA* promoter activity observed at 30°C.

### Identification of an expanded putative Vp ExsA binding motif for 11 genetic loci

We noted that elevated Vpa0450 and VopN secretion levels exclusively correlated with growth conditions that supported elevated *exsA* gene expression (Figures 2, 3). This observation is in direct agreement with a RNA-seq study showing ExsA overexpression upregulated *vpa0450* and *vopN* gene expression (Nydam et al., 2014). Therefore, we sought to discover an explanation that could link elevated Vpa0450 and VopN protein secretion to *exsA* expression.

**Figure 8:**
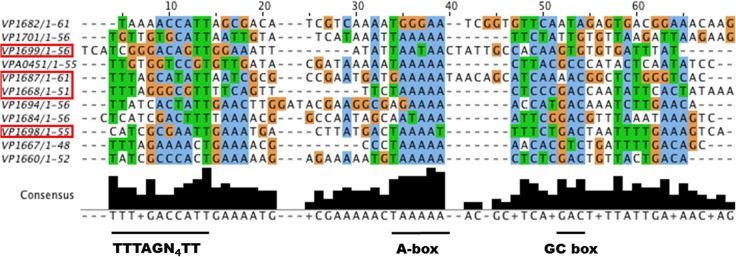
**A putative expanded ExsA binding motif is found for many Vp T3SS-1 associated genes**. Vp T3SS-1 promoter elements were retrieved from the genome sequence of Vp RIMD2210633 within the NCBI database, aligned using MUSCLE (Edgar, 2004), and visualized with JALView (Waterhouse et al., 2009). Boxed genes have been validated for either ExsA binding or ExsA mediated gene regulation in this study. The underlined regions below the sequences represent conserved DNA motifs. The TTTAGN_4_TT motif has been shown to support ExsA binding to DNA in Vp. The A-box and GC box motifs are known to support DNA binding for *P. aeruginosa* ExsA.

We hypothesized that ExsA could activate *vpa0450* and *vopN* gene expression by recognizing the respective promoter regions of these genes. For Vp, a previous study using two promoter DNA fragments identified an ExsA DNA binding motif (TTTAGN_4_TT) (Zhou et al., 2008). This DNA binding motif is similar to one identified for VirF, an AraC-type regulator of *Yersinia* species (Wattiau and Cornelis, 1994). Notably, Vp *exsA* has been shown to complement a *P. aeruginosa exsA* mutant for transcriptional activation (King et al., 2013) and detailed mutagenesis studies have implicated an A-box and a separate G/C box motif in *P. aeruginosa* ExsA binding to DNA (King et al., 2012). Therefore, we predicted that the identified DNA motifs for both Vp and *P. aeruginosa* ExsA DNA binding would be present as a “hybrid” or expanded motif upstream of Vp *exsA*-dependent promoters. Sequence analysis of the DNA region upstream of *vopN (vp1667)* and *vpa0451* (a chaperone gene co-transcribed with *vpa0450*), revealed DNA sequences consistent with our prediction. Specifically, considerable identity to the binding motif (TTTAGN_4_TT), along with an A-box and a G/C box (Figure 8).

Next, we considered that the observed increase in T3SS-1 associated proteins and effectors under LB+Mg+EGTA growth conditions (Figure 6) likely required increased expression of specific T3SS-1 apparatus genes, presumably achieved through ExsA. We evaluated all the annotated T3SS-1 genes and identified 11 putative expanded ExsA binding sites (Figure 8). Two of these are known to bind ExsA (*vp1668* and *vp1687*) and we provide evidence in this study that *exsA (vp1699)* and *exsD (vp1698)* are regulated by ExsA. Putative expanded ExsA binding sites are located upstream of chaperone and effector gene pairs (*vp1684-vp1683, vp1687-vp1686, vp1682-vp1680*).

## Discussion

In this study, we profiled two distinct promoter elements that control *exsA* gene expression. Specific growth conditions produced differential *exsA* promoter activity. Maximal *exsA* gene expression correlated with elevated T3SS-1 protein secretion levels when Vp was grown in media supplemented with Mg+EGTA at 30°C. In contrast, *exsA* gene expression and T3SS-1 activity was minimal (baseline) when Vp was cultured in the presence of calcium. We identify that ExsA serves as an auto-activator, while at the same time positively regulates gene expression of *exsD*, an inhibitor of ExsA activity. Lastly, combining known features of DNA binding motifs from Vp ExsA, *P. aeruginosa* ExsA, and *Yersinia* VirF, fresh bioinformatic analyses identified a putative expanded Vp ExsA DNA binding motif linked to multiple T3SS-1 genetic operons. The findings indicate that Vp ExsA is a key entry level regulator of T3SS-1 gene expression and that multiple genetic and environmental factors contribute to *exsA* gene expression levels.

Genetic analyses have identified a transcriptional start site and putative promoter upstream of *exsA* (Sun et al., 2015). In *V. harveyi*, an *exsBA* co-transcript has been identified (Waters et al., 2010), which is somewhat surprising given the large *exsB*-*exsA* intergenic region [~600bp, also present in Vp (Figure 1)]. This prompted us to investigate whether *exsBA* are co-transcribed in Vp. Using RT-PCR, we detected a Vp *exsBA* transcript (Figure 1). This finding indicates that *exsA* gene expression can be initiated by two distinct promoter elements, similar to *V. harveyi*. Quantitative activity profiling of the distinct *exsB* and *exsA* promoters revealed differential temporal features and activity levels (Figure 3). Collectively, the promoter profiling data indicates that for the growth conditions tested, Vp can initiate *exsBA* co-transcription followed by high level *exsA* gene expression from a proximal promoter.

A clear finding from this study is that high T3SS-1 secreted protein levels correlate with an increase in *exsA* promoter activity (Figures 6, 7). We had previously shown that growth media with calcium reduced Vp T3SS-1 activity and that Mg+EGTA growth media promoted T3SS-1 activity (Sarty et al., 2012), although the mechanisms behind those observations were not evident. Multiple growth conditions were used to profile light emission of an *exsA* promoter-lux fusion which indicated that when Vp is grown in the presence of calcium, *exsA* promoter activity (and by extension *exsA* gene expression) was at low baseline levels. In sharp contrast, Vp growth in Mg+EGTA significantly upregulated *exsA* promoter activity. Hence, growth conditions can impact *exsA* gene expression levels with consequences for T3SS-1 activity.

Temperature is known to have a regulating impact on gene expression and T3SS activity for many pathogens. In the case of Vp, it appears that *exsA* gene expression is elevated at 30°C compared to 37°C (Figure 7). This observation extends to protein secretion levels where translocon and effectors were relatively more abundant at 30°C. It is important to note that T3SS-1 associated proteins are expressed and secreted at 37°C, which agrees with various lines of evidence from cytotoxicity and infection assays (Park et al., 2004a; Broberg et al., 2010; Zhou et al., 2010b; Sarty et al., 2012). While T3SS-1 is required for full virulence in a small animal model of infection (Ritchie et al., 2012), it's role in human diarrheal disease has yet to be confirmed. A recent transcriptomic screen of Vp during infection suggests that ExsA levels need to reach a threshold to mediate downstream events (Livny et al., 2014). Our data supports this view, particularly with respect to differential *exsA* promoter activation.

The data indicate that *in vitro* low calcium conditions are associated with elevated *exsA* promoter activation. This is similar to many observations from different pathogens that express a T3SS, including *Yersinia, Pseudomonas*, and EPEC (DeBord et al., 2003; Deng et al., 2005; Dasgupta et al., 2006). However, we are cautious with our interpretations since the experiments used EGTA, which can chelate a variety of divalent cations with varying efficiency. The affinity of EGTA for calcium is known to be high, although other cations can be bound including Fe^2+^. We also note that the inclusion of calcium to Vp growth media containing magnesium and EGTA was sufficient to limit *exsA* promoter activity (Figure 2), although one cannot rule out the possibility that other chelated cations are freed up due to EGTA competitively binding calcium. For example, low soluble iron availability has been implicated in Vp T3SS induction (Gode-Potratz et al., 2010). Under certain *in vitro* conditions tested here, soluble iron cations may have come available when EGTA competitively bound to calcium, potentially limiting T3SS-1 activity. Therefore, it remains to be determined what exact environmental signals induce Vp T3SS-1, although low amounts of free divalent cations could be involved. During Vp infection of human cells, bacterial contact with target cells also is believed to have a role in T3SS-1 induction (Zhou et al., 2008), although the detailed mechanisms have not been elucidated to date.

A secretion hierarchy in T3SS occurs with translocator proteins being secreted ahead of effector proteins (Tomalka et al., 2012). Interestingly, different total secreted protein profiles were observed when Vp was cultured in specific growth media (Figure 6). The inclusion of Mg+EGTA to LB resulted in higher levels of effectors along with the specific appearance of VopN and Vpa450 effector proteins in secreted fractions. *exsA* promoter activity levels were significantly different for LB+Mg and LB+Mg+EGTA conditions especially at 3 h post inoculation (Figure 2). This prompted us to examine if ExsA could be involved in activating gene expression of *vopN* and *vpa0450*. In both cases, we identified DNA sequences that strongly match an expanded Vp ExsA binding motif (containing TTTAGN4TT, A-box, and G/C box motifs) located upstream of these genes (Figure 8). Furthermore, our bioinformatic analyses reveal putative expanded ExsA binding motifs upstream of 11 genetic loci associated with T3SS-1 operons. Two of these genetic loci have been shown to support ExsA binding, and we provide promoter activity data to implicate the *exsA* and *exsD* promoters as being regulated by ExsA (Figure 5).

The 11 identified genetic loci in Figure 8 are arranged in transcriptional operons and collectively encode 41 genes. This number of ExsA regulated genes is in agreement with a recent Vp RNA-Seq study (Nydam et al., 2014) and also closely resembles the 10 ExsA regulated operons within *P. aeruginosa* (Diaz et al., 2011). The expanded Vp ExsA binding motif identified here combines DNA elements that bring together data from various systems in *Yersinia* (VirF), *Pseudomonas* (ExsA) and Vp (ExsA) (Wattiau and Cornelis, 1994; Brutinel et al., 2008; Zhou et al., 2008). The fact that Vp *exsA* can complement a *P. aeruginosa exsA* mutant (King et al., 2013) further indicates common functionality at a DNA binding level. The binding of AraC/XylR/ExsA family members to DNA is complex, in some cases involving sequential steps. Therefore, we propose that further DNA binding assays and detailed mutagenesis investigations are required to experimentally validate and delineate the expanded Vp ExsA binding motif presented in Figure 8.

In summary, Vp was found to have two distinct mechanisms to initiate *exsA* expression. Growth media and temperature were found to impact on T3SS-1 activity, which was linked to *exsA* expression levels. ExsA auto-activates is own expression, whereas its negative regulator ExsD is expressed under similar conditions. ExsA is therefore a central transcriptional activator that responds to environmental stimuli to regulate T3SS-1 associated genes. The results suggest that temporal ExsA cellular levels exhibit fine tuning regulatory roles resulting in differential T3SS-1 activity.

### Conflict of interest statement

The authors declare that the research was conducted in the absence of any commercial or financial relationships that could be construed as a potential conflict of interest.

## Supplementary material

Figure S1 **(A)** *exsA* promoter activity profiles within Δ*vscN* grown in LB (−) or LB+Mg+EGTA (+). **(B)** *exsB* promoter activities for various growth media conditions. Samples were measured at 2.5 h post inoculation. A representative data set is shown.

## References

[B1] Banerjee S. Petronella N. Chew Leung C. Farber J. (2015). Draft Genome Sequences of Four *Vibrio parahaemolyticus* Isolates from Clinical Cases in Canada. Genome Announc 3:e01482-14. 10.1128/genomeA.01482-14 25635013 PMC4319507

[B2] Broberg C. A. Zhang L. Gonzalez H. Laskowski-Arce M. A. Orth K. (2010). A Vibrio effector protein is an inositol phosphatase and disrupts host cell membrane integrity. Science 329, 1660–1662. 10.1126/science.1192850 20724587

[B3] Brouwers E. Ma I. Thomas N. A. (2012). Dual temporal transcription activation mechanisms control cesT expression in enteropathogenic Escherichia coli. Microbiology 158(Pt 9), 2246–2261. 10.1099/mic.0.059444-0 22723287

[B4] Brutinel E. D. Vakulskas C. A. Brady K. M. Yahr T. L. (2008). Characterization of ExsA and of ExsA-dependent promoters required for expression of the Pseudomonas aeruginosa type III secretion system. Mol. Microbiol. 68, 657–671. 10.1111/j.1365-2958.2008.06179.x 18373522

[B5] Brutinel E. D. Vakulskas C. A. Yahr T. L. (2009). Functional domains of ExsA, the transcriptional activator of the Pseudomonas aeruginosa type III secretion system. J. Bacteriol. 191, 3811–3821. 10.1128/JB.00002-09 19376850 PMC2698394

[B6] Ceccarelli D. Hasan N. A. Huq A. Colwell R. R. (2014). Distribution and dynamics of epidemic and pandemic *Vibrio parahaemolyticus* virulence factors. Front. Cell. Infect. Microbiol. 3:97. 10.3389/fcimb.2013.00097 24377090 PMC3858888

[B7] Dasgupta N. Ashare A. Hunninghake G. W. Yahr T. L. (2006). Transcriptional induction of the Pseudomonas aeruginosa type III secretion system by low Ca2+ and host cell contact proceeds through two distinct signaling pathways. Infect. Immun. 74, 3334–3341. 10.1128/IAI.00090-06 16714561 PMC1479281

[B8] DeBord K. L. Galanopoulos N. S. Schneewind O. (2003). The ttsA gene is required for low-calcium-induced type III secretion of Yop proteins and virulence of Yersinia enterocolitica W22703. J. Bacteriol. 185, 3499–3507. 10.1128/JB.185.12.3499-3507.2003 12775686 PMC156212

[B9] Deng W. Li Y. Hardwidge P. R. Frey E. A. Pfuetzner R. A. Lee S.. (2005). Regulation of type III secretion hierarchy of translocators and effectors in attaching and effacing bacterial pathogens. Infect. Immun. 73, 2135–2146. 10.1128/IAI.73.4.2135-2146.2005 15784556 PMC1087438

[B10] Diaz M. R. King J. M. Yahr T. L. (2011). Intrinsic and extrinsic regulation of type III secretion gene expression in pseudomonas aeruginosa. Front. Microbiol. 2:89 10.3389/fmicb.2011.00089 21833328 PMC3153048

[B11] Edgar R. C. (2004). MUSCLE: multiple sequence alignment with high accuracy and high throughput. Nucleic Acids Res. 32, 1792–1797. 10.1093/nar/gkh340 15034147 PMC390337

[B12] Fields K. A. Plano G. V. Straley S. C. (1994). A low-Ca2+ response (LCR) secretion (ysc) locus lies within the lcrB region of the LCR plasmid in Yersinia pestis. J. Bacteriol. 176, 569–579. 8300512 10.1128/jb.176.3.569-579.1994 PMC205092

[B13] Gode-Potratz C. J. Chodur D. M. McCarter L. L. (2010). Calcium and iron regulate swarming and type III secretion in *Vibrio parahaemolyticus*. J. Bacteriol. 192, 6025–6038. 10.1128/JB.00654-10 20851895 PMC2976450

[B14] Henke J. M. Bassler B. L. (2004). Quorum sensing regulates type III secretion in Vibrio harveyi and *Vibrio parahaemolyticus*. J. Bacteriol. 186, 3794–3805. 10.1128/JB.186.12.3794-3805.2004 15175293 PMC419960

[B15] King J. M. Brutinel E. D. Marsden A. E. Schubot F. D. Yahr T. L. (2012). Orientation of Pseudomonas aeruginosa ExsA monomers bound to promoter DNA and base-specific contacts with the P(exoT) promoter. J. Bacteriol. 194, 2573–2585. 10.1128/JB.00107-12 22408167 PMC3347218

[B16] King J. M. Schesser Bartra S. Plano G. Yahr T. L. (2013). ExsA and LcrF recognize similar consensus binding sites, but differences in their oligomeric state influence interactions with promoter DNA. J. Bacteriol. 195, 5639–5650. 10.1128/JB.00990-13 24142246 PMC3889609

[B17] Kodama T. Gotoh K. Hiyoshi H. Morita M. Izutsu K. Akeda Y.. (2010a). Two regulators of *Vibrio parahaemolyticus* play important roles in enterotoxicity by controlling the expression of genes in the Vp-PAI region. PLoS ONE 5:e8678. 10.1371/journal.pone.0008678 20084267 PMC2800187

[B18] Kodama T. Yamazaki C. Park K. S. Akeda Y. Iida T. Honda T. (2010b). Transcription of *Vibrio parahaemolyticus* T3SS1 genes is regulated by a dual regulation system consisting of the ExsACDE regulatory cascade and H-NS. FEMS Microbiol. Lett. 311, 10–17. 10.1111/j.1574-6968.2010.02066.x 20722736

[B19] Laemmli U. K. (1970). Cleavage of structural proteins during the assembly of the head of bacteriophage T4. Nature 227, 680–685. 10.1038/227680a0 5432063

[B20] Livny J. Zhou X. Mandlik A. Hubbard T. Davis B. M. Waldor M. K. (2014). Comparative RNA-Seq based dissection of the regulatory networks and environmental stimuli underlying *Vibrio parahaemolyticus* gene expression during infection. Nucleic Acids Res. 42, 12212–12223. 10.1093/nar/gku891 25262354 PMC4231756

[B21] Macritchie D. M. Ward J. D. Nevesinjac A. Z. Raivio T. L. (2008). Activation of the Cpx envelope stress response down-regulates expression of several locus of enterocyte effacement-encoded genes in enteropathogenic Escherichia coli. Infect. Immun. 76, 1465–1475. 10.1128/IAI.01265-07 18227171 PMC2292881

[B22] Makino K. Oshima K. Kurokawa K. Yokoyama K. Uda T. Tagomori K.. (2003). Genome sequence of *Vibrio parahaemolyticus*: a pathogenic mechanism distinct from that of V cholerae. Lancet 361, 743–749. 10.1016/S0140-6736(03)12659-1 12620739

[B23] Meighen E. A. (1993). Bacterial bioluminescence: organization, regulation, and application of the lux genes. FASEB J. 7, 1016–1022. 8370470 10.1096/fasebj.7.11.8370470

[B24] Nydam S. D. Shah D. H. Call D. R. (2014). Transcriptome analysis of *Vibrio parahaemolyticus* in type III secretion system 1 inducing conditions. Front. Cell. Infect. Microbiol. 4:1. 10.3389/fcimb.2014.00001 24478989 PMC3895804

[B25] Ono T. Park K. S. Ueta M. Iida T. Honda T. (2006). Identification of proteins secreted via *Vibrio parahaemolyticus* type III secretion system 1. Infect. Immun. 74, 1032–1042. 10.1128/IAI.74.2.1032-1042.2006 16428750 PMC1360304

[B26] Park K. S. Ono T. Rokuda M. Jang M. H. Iida T. Honda T. (2004a). Cytotoxicity and enterotoxicity of the thermostable direct hemolysin-deletion mutants of *Vibrio parahaemolyticus*. Microbiol. Immunol. 48, 313–318. 10.1111/j.1348-0421.2004.tb03512.x 15107542

[B27] Park K. S. Ono T. Rokuda M. Jang M. H. Okada K. Iida T.. (2004b). Functional characterization of two type III secretion systems of *Vibrio parahaemolyticus*. Infect. Immun. 72, 6659–6665. 10.1128/IAI.72.11.6659-6665.2004 15501799 PMC523034

[B28] Ritchie J. M. Rui H. Zhou X. Iida T. Kodoma T. Ito S.. (2012). Inflammation and disintegration of intestinal villi in an experimental model for *Vibrio parahaemolyticus*-induced diarrhea. PLoS Pathog 8:e1002593. 10.1371/journal.ppat.1002593 22438811 PMC3305451

[B29] Sarty D. Baker N. T. Thomson E. L. Rafuse C. Ebanks R. O. Graham L. L.. (2012). Characterization of the type III secretion associated low calcium response genes of *Vibrio parahaemolyticus* RIMD2210633. Can. J. Microbiol. 58, 1306–1315. 10.1139/w2012-109 23145828

[B30] Sun F. Zhang Y. Qiu Y. Yang H. Yang W. Yin Z.. (2015). H-NS is a repressor of major virulence gene loci in *Vibrio parahaemolyticus*. Front. Microbiol. 5:675. 10.3389/fmicb.2014.00675 25566201 PMC4264476

[B31] Thomas N. A. Deng W. Puente J. L. Frey E. A. Yip C. K. Strynadka N. C.. (2005). CesT is a multi-effector chaperone and recruitment factor required for the efficient type III secretion of both LEE- and non-LEE-encoded effectors of enteropathogenic Escherichia coli. Mol. Microbiol. 57, 1762–1779. 10.1111/j.1365-2958.2005.04802.x 16135239

[B32] Tomalka A. G. Stopford C. M. Lee P. C. Rietsch A. (2012). A translocator-specific export signal establishes the translocator-effector secretion hierarchy that is important for type III secretion system function. Mol. Microbiol. 86, 1464–1481. 10.1111/mmi.12069 23121689 PMC3524397

[B33] Velazquez-Roman J. León-Sicairos N. de Jesus Hernandez-Diaz L. Canizalez-Roman A. (2014). Pandemic *Vibrio parahaemolyticus* O3:K6 on the American continent. Front. Cell. Infect. Microbiol. 3:110. 10.3389/fcimb.2013.00110 24427744 PMC3878053

[B34] Waterhouse A. M. Procter J. B. Martin D. M. Clamp M. Barton G. J. (2009). Jalview Version 2–a multiple sequence alignment editor and analysis workbench. Bioinformatics 25, 1189–1191. 10.1093/bioinformatics/btp033 19151095 PMC2672624

[B35] Waters C. M. Wu J. T. Ramsey M. E. Harris R. C. Bassler B. L. (2010). Control of the type 3 secretion system in Vibrio harveyi by quorum sensing through repression of ExsA. Appl. Environ. Microbiol. 76, 4996–5004. 10.1128/AEM.00886-10 20543047 PMC2916497

[B36] Wattiau P. Cornelis G. R. (1994). Identification of DNA sequences recognized by VirF, the transcriptional activator of the Yersinia yop regulon. J. Bacteriol. 176, 3878–3884. 8021169 10.1128/jb.176.13.3878-3884.1994 PMC205584

[B37] Zhou X. Konkel M. E. Call D. R. (2010a). Regulation of type III secretion system 1 gene expression in *Vibrio parahaemolyticus* is dependent on interactions between ExsA, ExsC, and ExsD. Virulence 1, 260–272. 10.3389/fcimb.2013.00110 21178451 PMC3073295

[B38] Zhou X. Konkel M. E. Call D. R. (2010b). Vp1659 is a *Vibrio parahaemolyticus* type III secretion system 1 protein that contributes to translocation of effector proteins needed to induce cytolysis, autophagy, and disruption of actin structure in HeLa cells. J. Bacteriol. 192, 3491–3502. 10.1128/JB.01493-09 20418402 PMC2897656

[B39] Zhou X. Shah D. H. Konkel M. E. Call D. R. (2008). Type III secretion system 1 genes in *Vibrio parahaemolyticus* are positively regulated by ExsA and negatively regulated by ExsD. Mol. Microbiol. 69, 747–764. 10.1111/j.1365-2958.2008.06326.x 18554322 PMC2610376

