# Supplementary figures and images for: Transcriptional profiling of Vibrio parahaemolyticus exsA reveals a complex activation network for type III secretion
Source: Front Microbiol. 2015 Oct 20;6:1089. doi: 10.3389/fmicb.2015.01089 (PMC4612142; doi:10.3389/fmicb.2015.01089)

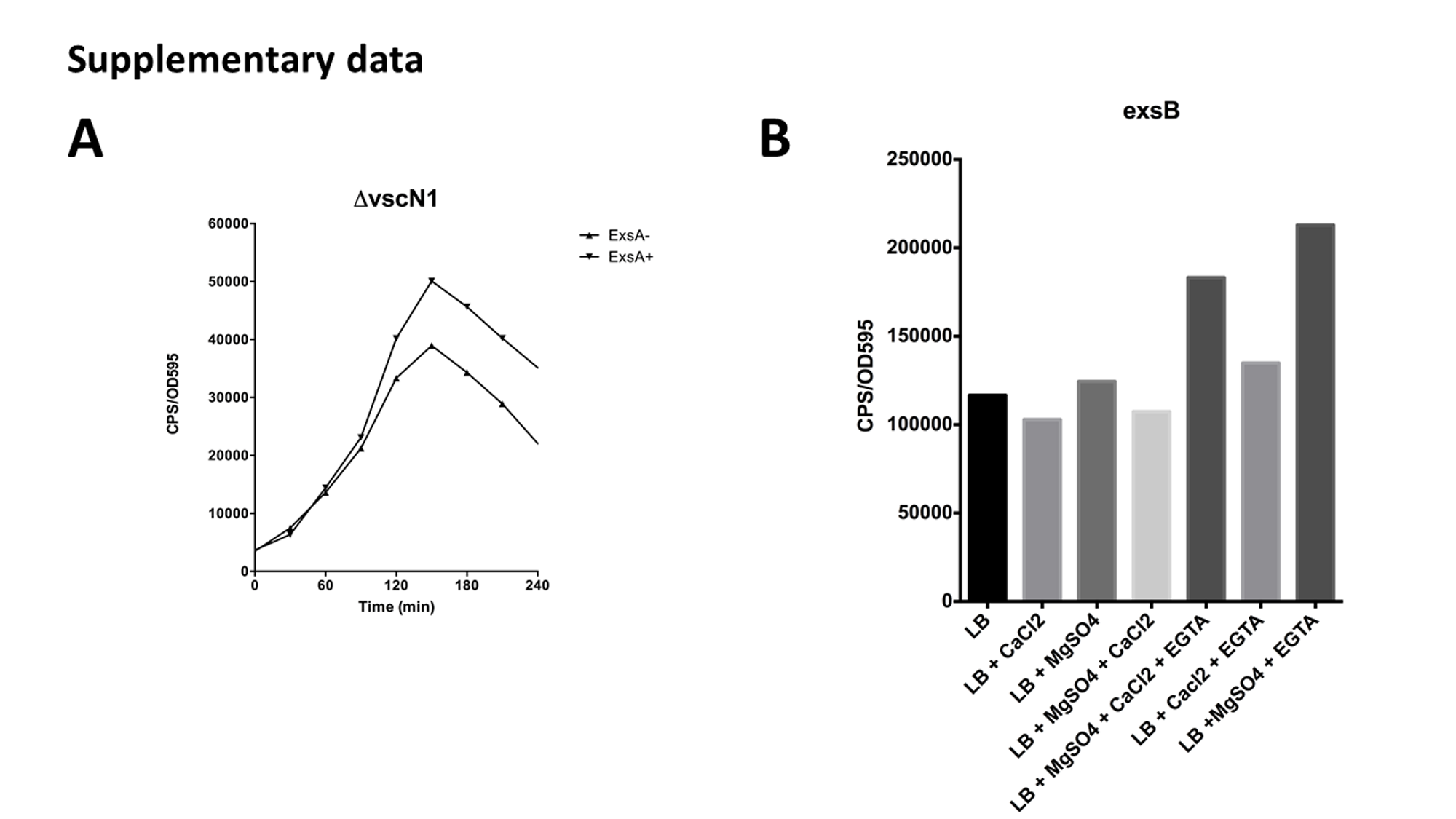

Supplement: Figure S1 — (A) exsA promoter activity profiles within ΔvscN grown in LB (−) or LB+Mg+EGTA (+). (B) exsB promoter activities for various growth media conditions. Samples were measured at 2.5 h post inoculation. A representative data set is shown. [file Image1.TIF]
